# Unlocking the Heart: A 10-Year Experience of Interventions and Outcomes of Constrictive Pericarditis in a Northeast Indian Tertiary Care Center

**DOI:** 10.7759/cureus.82369

**Published:** 2025-04-16

**Authors:** Reuben L Kynta, Sanjib Rawat, Rajeev Bharadwaj, Abhilash Goyal

**Affiliations:** 1 Department of Cardiothoracic and Vascular Surgery, North Eastern Indira Gandhi Regional Institute of Health and Medical Sciences, Shillong, IND; 2 Department of Cardiothoracic and Vascular Surgery, All India Institute of Medical Sciences, Guwahati, Guwahati, IND; 3 Department of Cardiology, All India Institute of Medical Sciences, Guwahati, Guwahati, IND; 4 Department of General and Minimal Access Surgery, All India Institute of Medical Sciences, Guwahati, Guwahati, IND

**Keywords:** chronic constrictive pericarditis, in-hospital mortality, mortality, northeast india, pericardiectomy, surgical intervention

## Abstract

Introduction: Chronic constrictive pericarditis (CCP) is a debilitating condition characterized by thickening and fibrosis of the pericardium, leading to impaired diastolic filling and reduced cardiac output. Patients typically present with symptoms of right-sided heart failure, such as peripheral edema, ascites, and dyspnea. Definitive treatment involves surgical pericardiectomy, which aims to remove the constrictive pericardium and restore normal cardiac function. This study aims to assess the operative and short-term outcome of this rare disease in the northeastern part of India.

Methods: Retrospective data records of patients who underwent pericardiectomy were analyzed from 2011 to 2020.

Results: Of the patients, 20 (47.6%) were in New York Heart Association (NYHA) class III, and 12 (28.6%) were in NYHA class IV. Hyperbilirubinemia was seen in 29 (69%) patients, and hypoalbuminemia in 18 (42.9%) patients. Radical pericardiectomy was done in 36 (85.7%) cases, and the waffle operation was done in five (12%) cases. The mean ICU stay post procedure was 5.81 days (2-18 days), and the mean hospital stay was 19.6 days (2-49 days). The in-hospital mortality rate was 14.6% (six cases), and the cause of death in all cases was persistent low cardiac output.

Conclusion: Pericardiectomy is the only definitive treatment for symptomatic CCP, and it gives a chance to the patient for a full recovery from the chronic morbidity and mortality it is associated with. The right time for surgical intervention is still not clear and needs to be individualized for each patient, but the earlier the intervention, the better the short-term survival.

## Introduction

Chronic constrictive pericarditis (CCP) is a rare but serious condition characterized by a thickened, fibrotic, and often calcified pericardium that restricts diastolic filling of the heart, leading to elevated venous pressures and reduced cardiac output. This condition can result from various etiologies, including idiopathic causes, viral infections, tuberculosis, previous cardiac surgery, and radiation therapy [1,2]. The hallmark pathophysiology of CCP is the impairment of ventricular filling due to the rigid pericardial sac, which leads to clinical manifestations such as progressive dyspnea, fatigue, ascites, and peripheral edema [3]. The demographic and clinical profile of this rare entity was crisply described in a study by the authors [4]. This study’s focus is on the management and short-term in-hospital morbidity and mortality.

Pericardiectomy, the surgical removal of the pericardium, is the definitive treatment for CCP. The procedure aims to relieve the constriction caused by the pericardial pathology, thereby improving cardiac filling, reducing symptoms, and enhancing overall quality of life [5]. While pericardiectomy is associated with significant perioperative risks, it remains the only curative option for patients with symptomatic constriction. The extent of pericardial resection, surgical approach, and timing of surgery are critical factors that influence patient outcomes [6]. Successful pericardiectomy can lead to dramatic improvements in functional status and survival, particularly when performed early in the disease course [7].

Despite advances in surgical techniques and perioperative care, the management of CCP remains challenging, particularly in cases where there is severe calcification or dense adhesions to adjacent structures. Understanding the indications, outcomes, and potential complications associated with pericardiectomy is essential for optimizing the treatment strategy for patients with CCP [8].

## Materials and methods

Retrospective data records of patients referred for pericardiectomy were analyzed. All patients underwent clinical and radiological workup. All patients with documented pathogenic infections received antibiotic therapy according to culture sensitivity prior to the primary or secondary surgical intervention.

Surgical method

**Figure 1:**
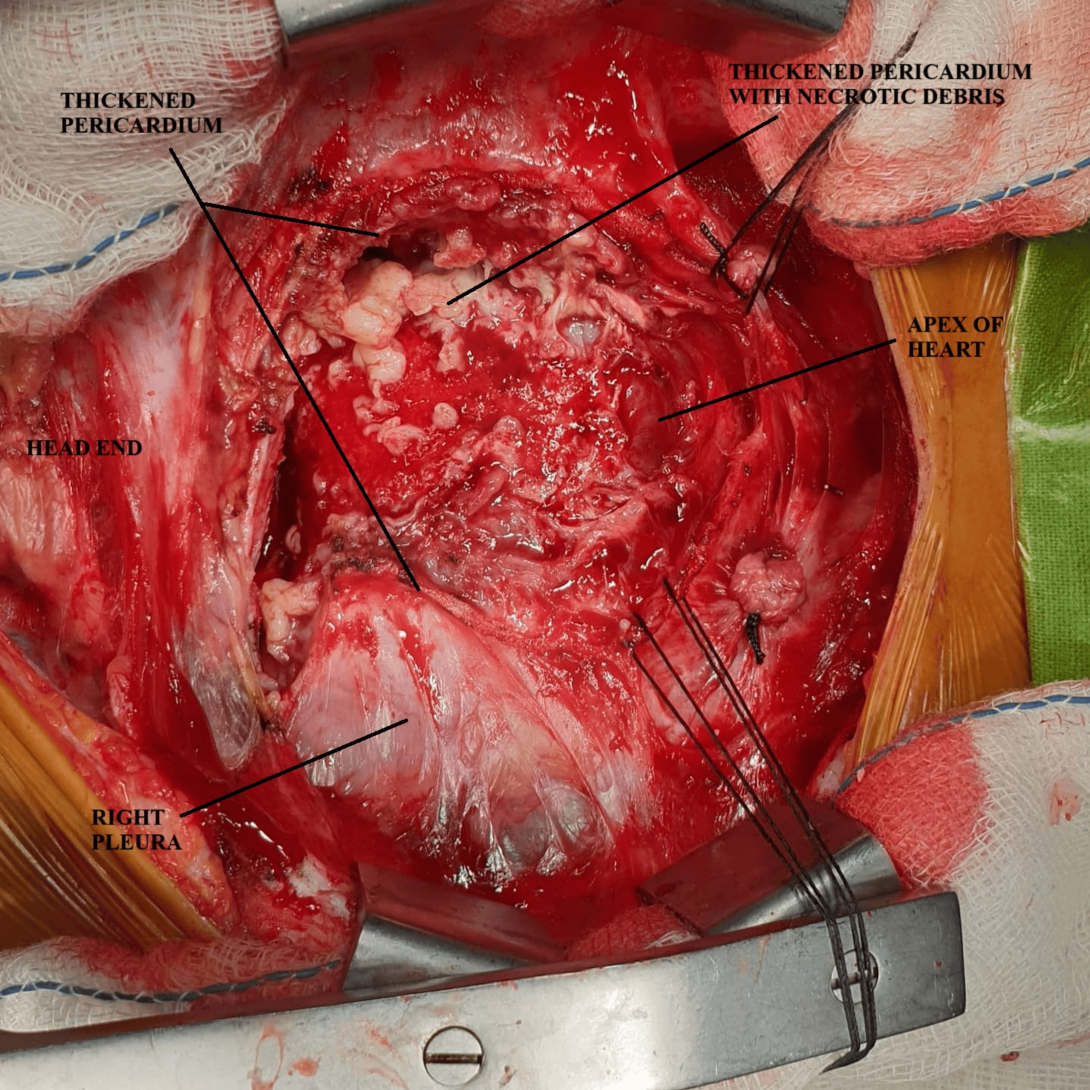
Intraoperative picture showing dissection of thickened parietal pericardium with caseous necrosis and thickened visceral pericardium. A small portion of the freed heart is seen near the apex.

All cases were performed via median sternotomy. After retracting the sternal halves, pericardial incision was made with a #15 surgical blade in cruciate patterns over the apex, right ventricle, and great arteries in a caudal cranial direction, and the corners were lifted with forceps (Figure 1).

**Figure 2:**
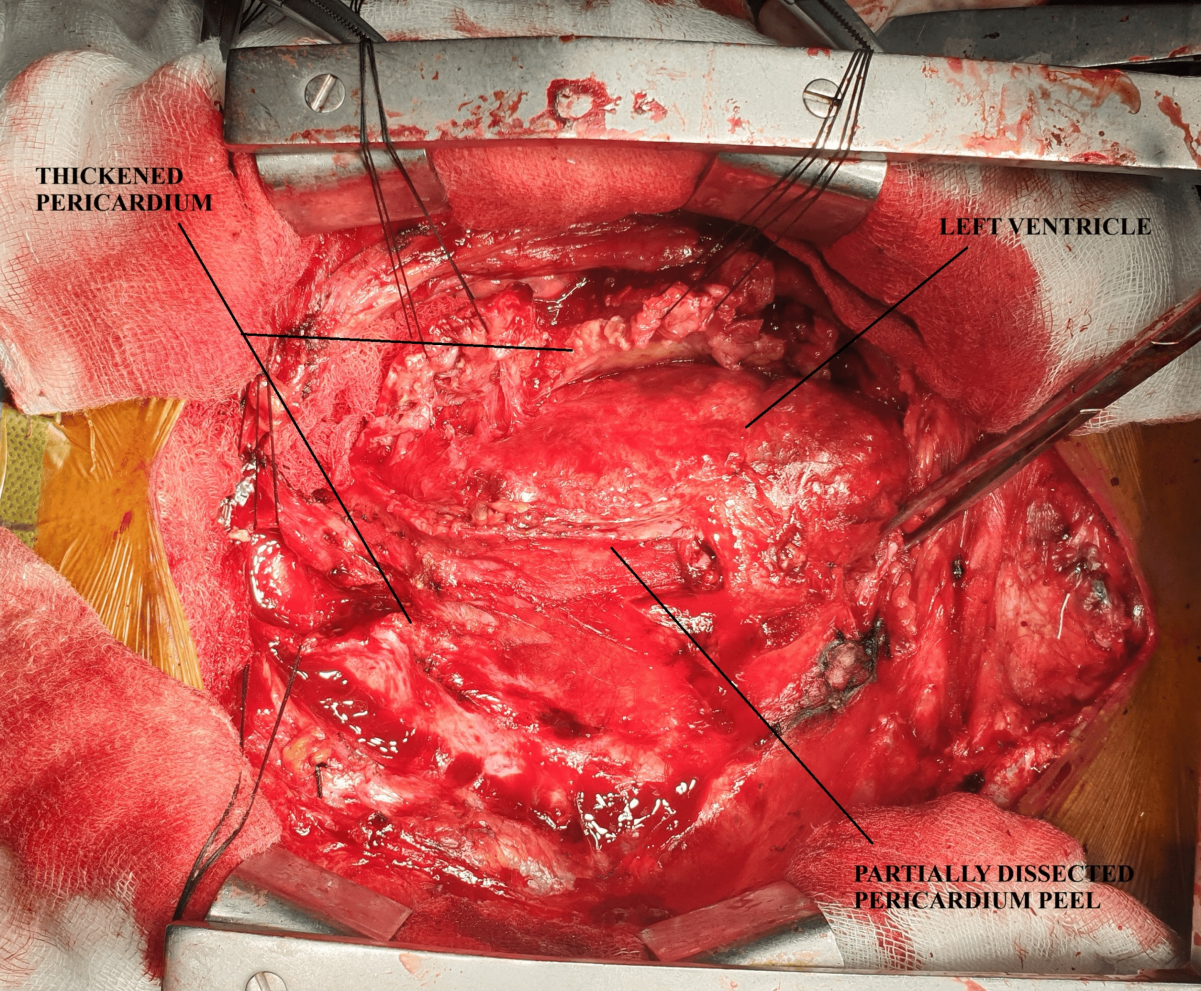
Intraoperative picture showing dissected pericardium over the outflow tract and left ventricle.

The epicardium was freed from the thickened visceral pericardium by scissors or peanut dissection. Areas of penetrating calcification deep into the myocardium were left in situ. Some areas of calcification were removed with the help of a Cavitron Ultrasonic Surgical Aspirator (CUSA; Sonopet, Stryker, Portage, MI). The left ventricle lateral wall was freed first, followed by the left half of the anterior pericardium covering the right ventricle and pulmonary trunk (Figure 2).

**Figure 3:**
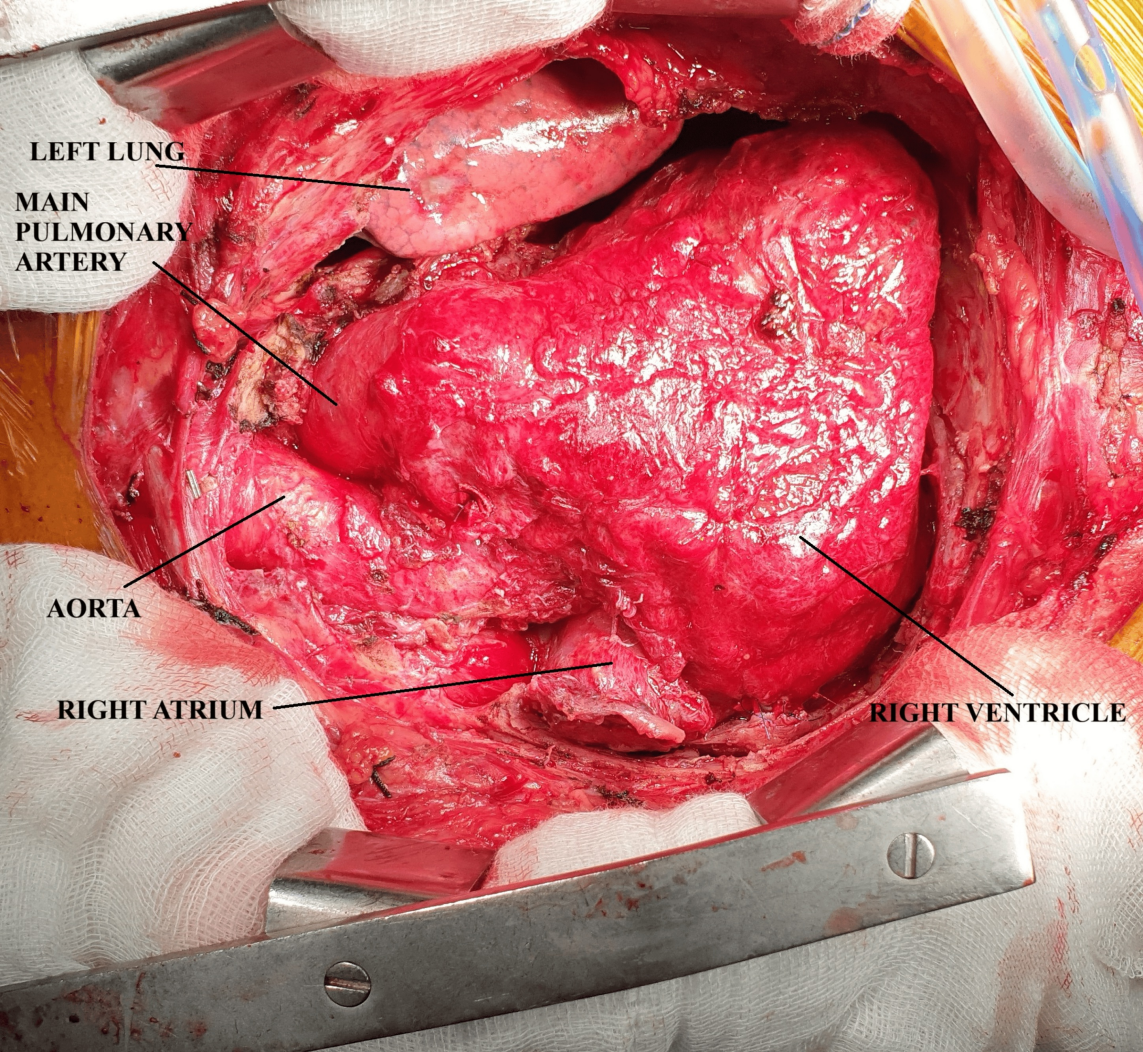
Intraoperative picture after complete pericardiectomy showing the freed heart.

The left half of the pericardium was then split into segments anterior to the left phrenic nerve, and silk stay sutures were used. The heart was then lifted cephalad for a short duration of 10-15 beats, and the muscular and membranous portions of the diaphragm were peeled from the posterior left ventricle and apex. The right atrium and great vena cava were then freed of constriction anterior to the right phrenic nerve (Figure 3).

In cases of extensive calcific constrictive pericarditis, a waffle operation was conducted.

Postoperative follow-up was performed till hospital stay. Analysis of variables in the preoperative and postoperative states was done.

Statistical study

All of the continuous variables are expressed via descriptive statistics, including frequency tabulation and categorical variables as percentages using SPSS version 30.0.0 (IBM Corp., Armonk, NY).

## Results

Preoperative variables

A total of 42 patients diagnosed with CCP who underwent anterior pericardiectomy were analyzed from 2011 to 2020. Males were more common (34, 81%), and the most common age group was less than 20 years of age, comprising 18 (42.9%) patients. The majority (47.6%) of patients were in New York Heart Association (NYHA) class III, and 28.6% were in NYHA class IV.

Hepatomegaly was seen in 22 (52.4%) patients, ascites in 15 (35.7%) patients, pleural effusion in 13 (31%) patients, and 29 (69%) patients had elevated jugular venous pressure. Pedal edema was seen in 36 (85.7%) patients. There was a prior history of anti-tubercular treatment in 22 (52.4%) patients. All patients received preoperative digoxin and diuretics.

Blood investigations revealed hyperbilirubinemia in 29 (69%) patients, with a mean bilirubin level of 2.04 mg/dl (range = 0.4-6.9). Hypoalbuminemia was seen in 18 (42.9%) patients, with a mean albumin level of 3.36 mg/dl (range = 2.1-4.1)

Transthoracic 2D echocardiography revealed septal bounce in 26 (61.9%) patients, pericardial thickening in all patients, with a mean thickness of 4.84 ± 3.04 mm, and calcification in 17 (40.5%) patients. Hepatic venous flow reversal was seen in 12 (28.6%), and moderate to severe tricuspid regurgitation was seen in 20 patients (47.6%). Preoperative variables are described in Table 1.

**Table 1:** Preoperative variables. NYHA: New York Heart Association. * Bharadwaj et al. (2024) [4].

Variables*	N (%)
Gender	
Male	34 (81%)
Female	8 (19%)
Age (years)	
<20	18 (42.9%)
21-40	13 (31.0%)
41-60	9 (21.4%)
>60	2 (4.8%)
NYHA status	
II	10 (23.8%)
III	20 (47.6%)
IV	12 (28.6%)
Clinical	
Hepatomegaly	22 (52.4%)
Ascites	15 (35.7%)
Pleural effusion	13 (31%)
Pedal edema	36 (85.7%)
Hyperbilirubinemia	29 (69%)
Hypoalbuminemia	18 (42.9%)
Preoperative digoxin and diuretics	42 (100%)
Echocardiogram	
Septal bounce	26 (61.9%)
Calcification	17 (40.5%)
Hepatic vein flow reversal	12 (28.6%)

Intraoperative variables

Radical pericardiectomy was done in 36 (85.7%) cases, and the waffle operation was done in five (12%) due to unfavorable anatomy and dense calcification in these cases. Of the cases, 40 (95%) were performed without cardiopulmonary bypass, whereas two (5%) cases were performed under cardiopulmonary bypass due to poor cardiac function.

Effusive constrictive pericarditis was seen in 13 patients. Pericardial calcification was present in 23 patients, caseous pockets in 30, and purulent membrane was present in 28 patients.

The mean ICU stay post procedure was 5.81 days (2-18 days), and the mean hospital stay was 19.6 days (2-49 days), as detailed in Tables 2, 3.

**Table 2:** ICU stay.

ICU stay	Frequency	Percentage
<10 days	38	90.5
>10 days	4	9.5
Total	42	100.0

**Table 3:** Hospital stay. Hospital stay includes total hospitalization duration from the operative day, including intensive care stay, till the time of discharge/death.

Hospital stay	Frequency	Percentage
<15 days	16	38.1
>15 days	26	61.9
Total	42	100.0

Postoperative variables

Histopathology revealed tuberculosis in 28 patients. In mortality analysis, the in-hospital mortality rate was 14.6% (six cases), of which four had NYHA class IV symptoms, and all of them had moderate to severe tricuspid regurgitation. The cause of death was persistent low cardiac output (Table 4).

**Table 4:** NYHA class outcome crosstabulation. NYHA: New York Heart Association. Outcome: Patients with NYHA class 4 preoperative symptoms had the highest mortality. * Bharadwaj et al. (2024) [4].

NYHA class (Preoperative)	Outcome (Postoperative)		Total*
	Alive	Expired	
II	10	0	10
III	18	2	20
IV	8	4	12
Total	36	6	42

## Discussion

Complete pericardiectomy for CCP can be achieved with or without cardiopulmonary bypass. The use of cardiopulmonary bypass achieves a more thorough and complete resection of the pericardium, with the limitations of additional bypass time and cost.

All patients in our study underwent pericardiectomy or the waffle operation in case of extensive calcification. Without extracorporeal oxygenation and left ventricular ventilation, pericardial constriction is not effectively eliminated [9,10], and there is a risk of postoperative low cardiac output syndrome [11]. In-hospital mortality in our study was 14.6% (six cases out of 42), which was higher than 3.9% (two cases out of 51) in a Chinese five-year single-center experience [12]. Another study from Spain had an in-hospital mortality of 16% (five cases out of 31) [13]. The reason for high mortality in our study may be due to the more symptomatic (class IV symptoms) and higher grade of tricuspid regurgitation.

In-hospital mortality has been linked to factors such as advanced age, prolonged symptom duration, higher functional class, atrial fibrillation, moderate to severe tricuspid regurgitation, left ventricular dysfunction, kidney failure, hyponatremia, hyperbilirubinemia, and elevated right atrial pressure [14].

This brings an important point on the right time for referral for surgery, which in the current literature is still unclear. Currently, most experts agree on referral for pericardiectomy in symptomatic patients who are intolerant or not responding to medications, especially diuretics. Individualized decision for each patient is still the norm rather than a cutoff of NYHA class symptoms or response to medications. As the disease process is chronic, referral for surgery is late most of the time, especially in our part of the country.

In terms of surgical approach, there was no difference found between anterolateral thoracotomy versus median sternotomy approach in one study [15]. In our center, we prefer median sternotomy in all cases because of greater clearance of adhesions, ease for conversion to cardiopulmonary bypass if need arises, and also due to the presence of pleural adhesions, as most of the cases were tubercular in origin, which would require decortication.

Limitations

Our study, being a retrospective analysis, had the inherent biases of study design and incomplete documentation associated with a retrospective analysis. The sample size being small also limits the generalizability of the data to the whole region. The lack of long-term follow-up hinders the assessment of survival rates of this rare entity.

## Conclusions

In conclusion, CCP remains a significant cause of morbidity in Northeast India, particularly due to the high prevalence of tuberculosis, a leading etiology in the region. Surgical intervention, primarily pericardiectomy, is the definitive treatment and has shown promising outcomes in alleviating symptoms and improving cardiac function. However, postoperative morbidity and mortality rates can be influenced by factors such as the duration of disease, severity of constriction, and comorbid conditions. In resource-limited settings like Northeast India, delayed diagnosis and limited access to specialized surgical care may exacerbate outcomes. Despite these challenges, timely surgical intervention, combined with improved perioperative care and patient follow-up, can significantly reduce morbidity and enhance quality of life. Addressing barriers to early diagnosis and treatment, along with raising awareness about CCP, is crucial for improving long-term prognosis in this region.
